# Variability in clinical assessment of clade IIb mpox lesions

**DOI:** 10.1016/j.ijid.2023.10.004

**Published:** 2023-12

**Authors:** Benjamin Jones, Amy Paterson, Naseem AlKhoury, Josephine Bourner, Jake Dunning, Piero Olliaro, Amanda Rojek

**Affiliations:** Pandemic Sciences Institute, University of Oxford, Oxford, United Kingdom

**Keywords:** Mpox, Agreement, Reliability, Lesion assessment

## Abstract

**Objectives:**

The ongoing global outbreak of mpox, caused by clade IIb mpox virus, poses significant challenges in accurately categorizing and assessing the diversity of lesions. With lesion resolution being a key endpoint in clinical trials and observational studies, it is essential to evaluate the inter-rater reliability and agreement of clade IIb mpox lesion assessment among clinicians.

**Methods:**

Clinicians experienced in clade IIb mpox disease were surveyed online with 20 lesion images. They categorized lesions into active, crusted, resolved, or unclassifiable groups. Reliability was assessed with Fleiss' kappa and agreement with proportion of exact agreement.

**Results:**

Fifty-three clinicians completed the survey, with a median self-reported confidence rating of 7 (on a scale of 1 to 10) in assessing mpox lesions. The inter-rater reliability was found to be moderate, with a Fleiss' kappa coefficient of 0.417 (*P* <0.05, 95% confidence interval: 0.409-0.425). The inter-rater agreement was 61%.

**Conclusion:**

This study demonstrates moderate inter-rater reliability and agreement in clade IIb mpox lesion assessment among clinicians. The findings emphasize the importance of standardizing lesion classification systems to facilitate clinical care (e.g., decision to start treatment) and public health (e.g. isolation) decisions and a need to explore alternative endpoints for clinical trials.

## Highlights

• There are challenges in categorizing mpox lesions.
• We assessed the inter-rater reliability and agreement among clinicians.
• We found moderate reliability and agreement in mpox lesion assessment.
• This study has implications for interpreting trial results.
• There is a need for standardized lesion classification in clinical trials.

## Introduction

Mpox (formerly known as monkeypox) is an infectious disease caused by the mpox virus. The 2022-2023 global outbreak of mpox is caused by clade IIb mpox virus - the focus of this study [1].

Clade IIb mpox clinical presentation differs from previous outbreaks in Africa (clade I/IIa disease) with a predominance of perianal and genitourinary lesions, new complications such as proctitis, and lower mortality [2]. There is a broader spectrum of lesions that defy the classification systems previously used for Orthopoxviruses.

Classifying whether a lesion is active or resolved is important for clinical decisions about treatment and public health decisions about self-isolation. Accurate assessment is also critical for research [2]. Several clinical trials have been launched worldwide to investigate potential treatments. These all use lesion resolution as their primary endpoint. Urgent investigation is therefore warranted to ensure that clinical trials and clinical decision-making are based on a robust endpoint. This study is the first to assess variability in assessment of mpox lesions at the clinical interface.

## Methods

This study was conducted between March and May 2023. The Guidelines for Reporting Reliability and Agreement Studies (GRRAS) were used [3]. The study was granted ethical approval from the University of Oxford (R84355/RE001).

### Survey design

Twenty anonymized lesion images were imported from the literature and under the Creative Commons Attribution License or Creative Commons Attribution Non-Commercial License. Images showing a range of lesion types, body locations, and skin colors were selected and reviewed by a recognized expert in mpox clinical management to ensure adequate range and clarity of images. The final survey can be seen in Appendix A.

### Participants

The inclusion criteria were clinicians from the United Kingdom and European Union working in the fields of infectious diseases, emergency medicine, internal medicine, dermatology, general practice, or sexual health who had managed clade IIb human mpox virus disease. The exclusion criteria were clinicians not practicing clinically or completing their internship or foundation years.

### Data collection process

Participants were presented with 20 slides, each containing a single image of a clade IIb mpox lesion. The participants were asked to categorize each lesion independently, choosing one of the following options: active, crusted and scabbed, resolved and healed, or unable to classify. Participants were provided with the World Health Organization's working definition of each of these categories [4]. Participants had 30 minutes to complete the questionnaire and could only complete it once. Each clinician's specialty, country of practice, estimated number of mpox patients seen, self-rated confidence in assessing mpox lesions, and perception of the availability of clinical guidelines for managing mpox patients were sought. Participants did not receive training for the study.

### Data analysis

All analyses were performed using R Statistical Software (v4.1.2; R Core Team 2021). Categorical variables were described as proportions. Continuous variables were described as mean and standard deviation if normally distributed or median and inter-quartile range (IQR) if not normally distributed. Inter-rater reliability was measured using Fleiss’ kappa coefficient due to its suitability for nominal data and studies that involve multiple raters [5]. The ‘irr’ package in R used for calculating Fleiss’ kappa was unable to calculate the confidence interval, therefore it was generated through bootstrapping using the ‘raters’ package and Monte Carlo simulation. Inter-rater agreement was measured using proportion of exact agreement. The levels of agreement across all lesions were plotted to investigate clinicians’ agreement patterns. A range of sensitivity analyses were conducted to assess the robustness of the findings (see Appendix B for these methods).

## Results

### Description of participants

A total of 109 potential participants received and opened the survey, 53 of whom completed the survey. All participants who started the survey completed it. A description of the participant's specialism, country of practice, number of mpox patients seen, and feelings towards adequacy of available guidelines for managing mpox are shown in Table 1. On a scale for self-reported confidence assessing mpox patients on a scale of one (no confidence) to ten (complete confidence) the median confidence rating was 7 (IQR: 5-8) (Appendix C for distribution).

**Table 1:** Description of study participants.

Variable	Number of participants (%), N = 53
Specialist field	
Dermatology	1 (2)
Genitourinary medicine	3 (6)
HIV medicine	4 (8)
Infectious diseases	43 (81)
Internal medicine	2 (4)
Country of practice	
Belgium	1 (2)
France	35 (66)
Switzerland	1 (2)
Spain	1 (2)
United Kingdom	15 (28)
Number of mpox patients seen	
<5	16 (30)
5-10	7 (13)
10-20	9 (17)
20-50	13 (25)
>50	8 (15)
Perceived adequacy of clinical guidelines on mpox lesion assessment	
Adequate	25 (47)
Inadequate	14 (26)
Undecided	14 (26)

### Inter-rater reliability

The inter-rater reliability, of lesion assessments was 0.417 (*P* <0.05, z score = 107, 95% confidence interval = 0.409-0.425). The inter-rater reliability Fleiss’ Kappa values for each response category are as follows: ‘active lesion’ (Κ = 0.451, *P* <0.05), ‘crusted and scabbed lesion’ (Κ = 0.479, *P* <0.05), ‘resolved and healed lesion’ (Κ = 0.466, *P* <0.05), and ‘unable to classify’ (Κ = 0.079, *P* <0.05).

### Inter-rater agreement

**Figure 1:**
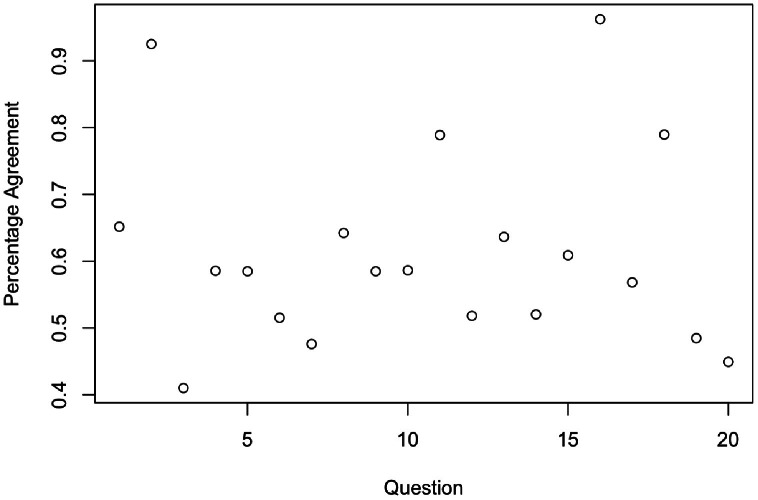
Proportion agreement for each question.

The exact proportion of agreement between the participants was found to be 61%. The proportion agreement per lesion question is shown in Figure 1.

Sensitivity analyses are described in Appendix B. These analyses included recalculating the inter-rater reliability after removing question 20 as it had a high proportion of 'unable to classify' responses, excluding individual participants one at a time to account for possible outlier participants, and restricting to clinicians who had seen more than 20 mpox patients in their clinical practice. It also included recalculating the inter-rater agreement removing all ‘unable to classify’ responses.

## Discussion

Clinicians with experience in treating mpox show only moderate agreement on classifying lesions based on single time-point photography of lesions. The proportion of exact agreement between the participants in this study was 61%. For the inter-rater reliability, the Fleiss Kappa was 0.417. This has clear implications for decisions regarding initiation of treatment, infection prevention and control procedures, and outcomes for clinical trials.

When expert clinicians are unable to agree on lesions despite the provision of a descriptive framework it creates a real challenge. Lesion assessment cannot be restricted to a very small subset of specifically trained dermatologists because this does not meet the reality of the broad range of treatment providers for a disease with over 80,000 cases. Furthermore, our anecdotal experience from lesion assessment working groups suggests that uncertainty continues even when decision-making is restricted to super-specialists. This will be an issue for the creation of ‘gold standard’ guidance. Some trials are using self-assessment of lesions from patients and the accuracy and reliability of this approach is not certain.

Interestingly, these findings occur despite participants’ relative confidence in their ability to assess mpox lesions (median = 7, IQR = 5-8). It is uncertain whether clinicians who feel less confident in their ability to classify lesions were less likely to fill in the questionnaire, and whether they would be more or less likely to agree with others’ assessment. Our sensitivity analysis, which we restricted to clinicians who had seen more than 20 mpox patients, suggests that those with more experience are more likely to agree but only minimally.

In terms of clinical assessment, lesion status is used to make decisions about whether to refer to specialist care, initiate or continue antiviral treatment, may contribute to decisions around hospitalization, and in some jurisdictions is the basis of guidance regarding self-isolation and infection prevention and control requirements. Disentangling the influence of lesion assessment on these decisions, compared with the myriad of other factors that contribute to a clinical decision is difficult, but clearly more robust and objective assessment options would assist with the development of clinical guidelines and help standardize infection prevention procedures. Selection of lesion resolution as an endpoint for clinical guidelines and trials was a very reasonable extrapolation from clade I mpox and smallpox (which show a more uniform progression of lesions) at the time, early during this outbreak, when they were developed. How to balance refinement of these outcomes as more data accumulates is a challenge. Clinical characterization protocols are critical, but waiting for the outcomes of these means that trials would potentially launch too late. In the interim, patients would continue to be provided treatment under compassionate care circumstances with minimal assessment of efficacy or safety. At the time of writing, no superior endpoint for clinical trials is obvious.

### Limitations

This study reports agreement rather than accuracy because there is no ‘correct answer’ to compare to. Assessment of a lesion based on a single photograph is likely to be inferior to assessment in real-life clinical settings, or assessment of a lesion over time where the evolution of the lesion can be taken into account. The study had a modest effective response rate of 49%. Most (81%) respondents were also from the same specialty (infectious disease physicians) who, at this point in the epidemic, tend to be responsible for the management of these patients in the locations of this study. This may affect the generalizability of the findings to other specialties managing clade IIb mpox disease. This may also reflect response bias because it is possible that clinicians with more expertise in mpox disease were more likely to participate. It is, therefore, plausible that the true inter-rater reliability and agreement might be lower than reported.

## Conclusion

This study assessed the inter-rater reliability and agreement of clade IIb mpox lesion assessment. We demonstrate moderate levels of reliability and agreement, providing valuable insights into the consistency of lesion categorization in clinical settings and the validity of lesion assessment for mpox as an endpoint in clinical trials.

## Declarations of competing interest

The authors have no competing interests to declare.

## Appendix Supplementary materials

Image, application 1

Image, application 2

Image, application 3
