# Supplementary material for: Variability in clinical assessment of clade IIb mpox lesions
Source: Int J Infect Dis. 2023 Dec;137:60–2. doi: 10.1016/j.ijid.2023.10.004 (PMC10914632; doi:10.1016/j.ijid.2023.10.004)
Supplement: Supplementary file 1 [file mmc1.docx]

# Human Mpox lesion assessment

Please choose the option you feel best describes the circled lesion for each of the following questions.

WHO working definitions are used for this assessment and are as follows:

Active lesion = Any of the following:

| Early lesions | Typically 1–3 mm that appear solid, are known as papules.Other lesions may develop a clear fluid-filled appearance known as a vesicle or, more often, will appear to contain white material (pustule/pseudo-pustule). |
| --- | --- |
| Progression | As lesions progress, central involution of the lesion may lead to a torus shape (umbilicated pseudo-pustule) that resembles a bagel or doughnut.A scale crust often develops first in the umbilicated central area of evolving skin lesions. |
| Late stage | Erosions and ulcers may also occur, which are also considered active.Superficial loss of a portion of the epidermis is an erosion, whereas full-thickness loss of the entire epidermis is an ulcer. |

Crusted and scabbed lesions = Evolving lesions may develop a surface of thick surface scale (stratum corneum), yellow serous crust, haemorrhagic crust or scab (eschar).Crusted lesions are considered infectious until the underlying lesion has completely resolved and the surface crust has been replaced by a full layer of epidermis.At this stage, most of the lesion should have crust without surrounding skin thickening (induration).

Resolved and healed lesions = Lesions are considered resolved once the lesion is no longer raised above the skin and any residual crust has resolved (desquamation).The underlying skin at the site of the lesion should appear intact.The skin may appear red or pink initially.Scar formation or “post-inflammatory” pigmentary changes (hyperpigmentation, hypopigmentation) may occur and persist for several weeks or longer after lesions have resolved.These “residual” changes are still considered a resolved/healed lesion.


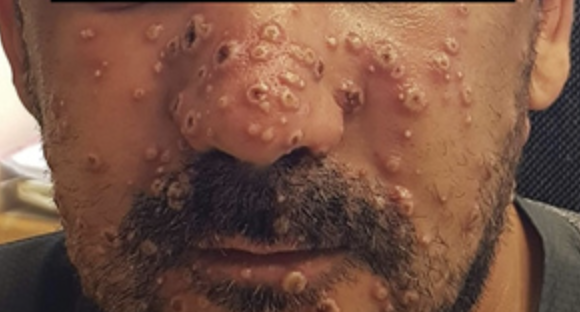


1.

a) active lesion (papules, vesicles, pustule/pseudo-pustule, umbilicated pseudo-pustule, erosion, or ulcer)

b) crusted and scabbed lesion (thick surface scale (stratum corneum), yellow serous crust, haemorrhagic crust or scab (eschar)).

c) resolved and healed lesion (lesion is no longer raised above the skin and any residual crust has resolved (desquamation)).

d) unable to classify


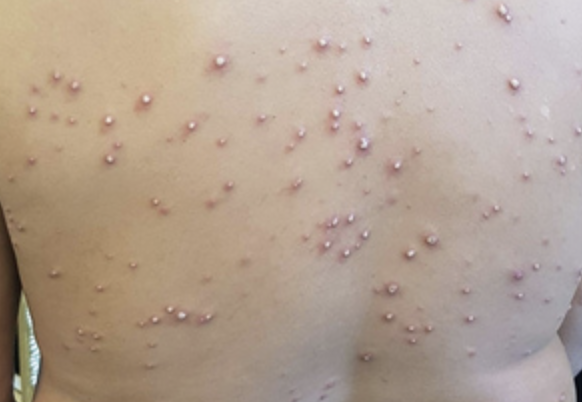


2.

a) active lesion (papules, vesicles, pustule/pseudo-pustule, umbilicated pseudo-pustule, erosion, or ulcer)

b) crusted and scabbed lesion (thick surface scale (stratum corneum), yellow serous crust, haemorrhagic crust or scab (eschar)).

c) resolved and healed lesion (lesion is no longer raised above the skin and any residual crust has resolved (desquamation)).

d) unable to classify

 
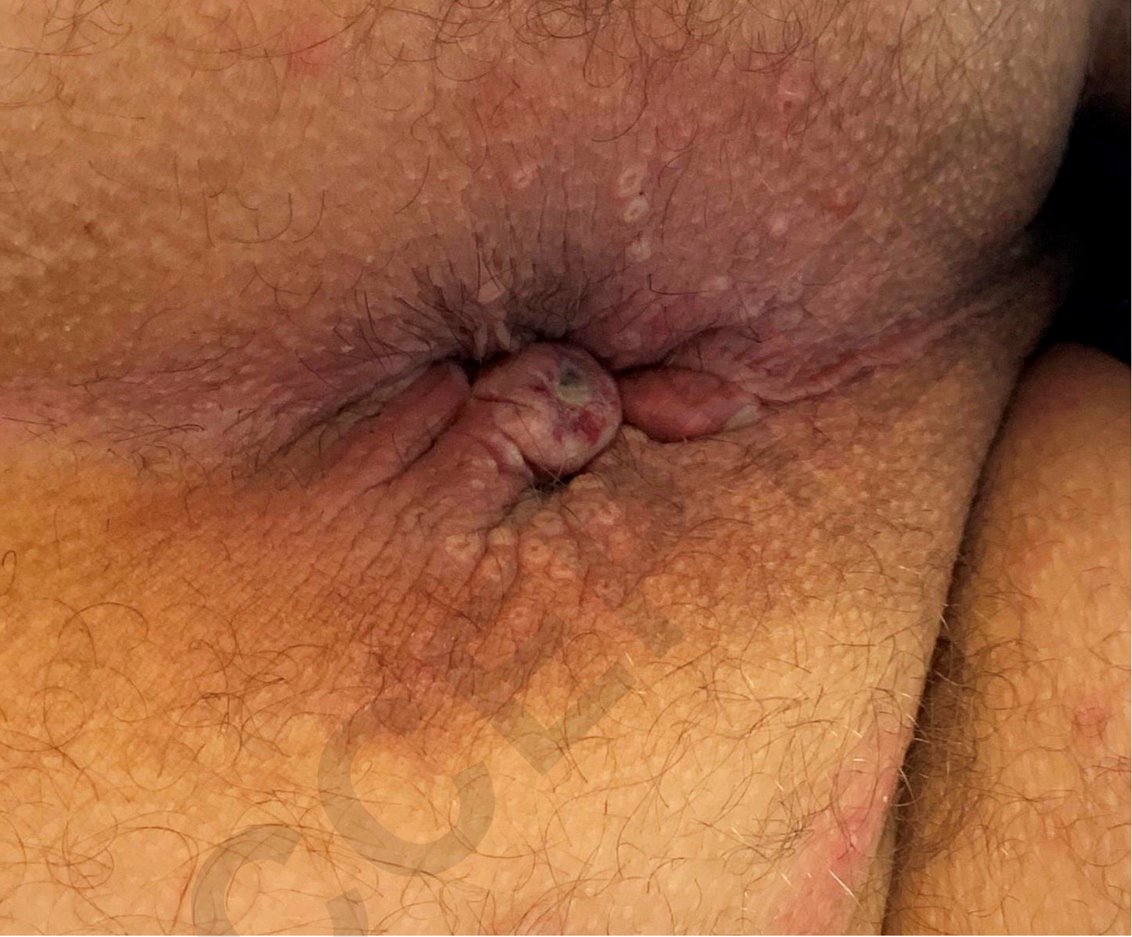


3.

a) active lesion (papules, vesicles, pustule/pseudo-pustule, umbilicated pseudo-pustule, erosion, or ulcer)

b) crusted and scabbed lesion (thick surface scale (stratum corneum), yellow serous crust, haemorrhagic crust or scab (eschar)).

c) resolved and healed lesion (lesion is no longer raised above the skin and any residual crust has resolved (desquamation)).

d) unable to classify


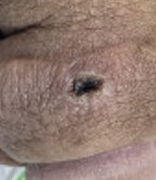


4.

a) active lesion (papules, vesicles, pustule/pseudo-pustule, umbilicated pseudo-pustule, erosion, or ulcer)

b) crusted and scabbed lesion (thick surface scale (stratum corneum), yellow serous crust, haemorrhagic crust or scab (eschar)).

c) resolved and healed lesion (lesion is no longer raised above the skin and any residual crust has resolved (desquamation)).

d) unable to classify

 
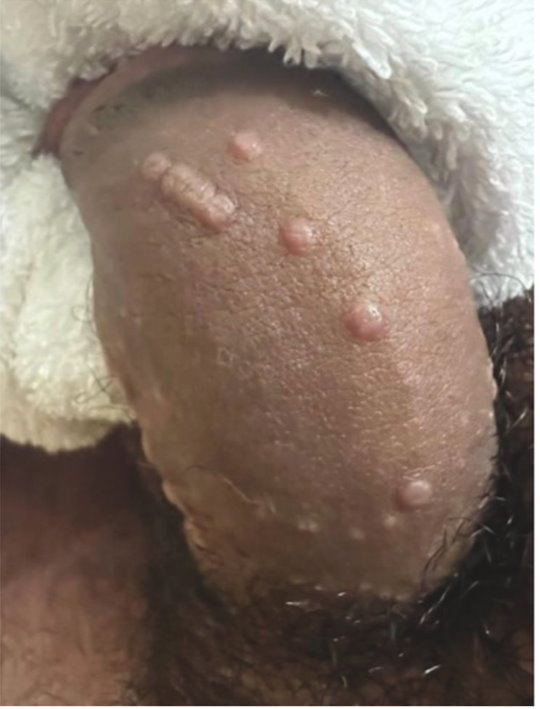


5.

a) active lesion (papules, vesicles, pustule/pseudo-pustule, umbilicated pseudo-pustule, erosion, or ulcer)

b) crusted and scabbed lesion (thick surface scale (stratum corneum), yellow serous crust, haemorrhagic crust or scab (eschar)).

c) resolved and healed lesion (lesion is no longer raised above the skin and any residual crust has resolved (desquamation)).

d) unable to classify


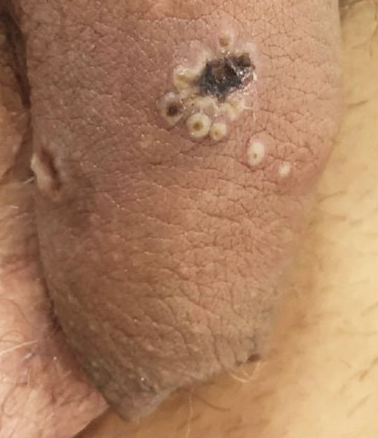


6.

a) active lesion (papules, vesicles, pustule/pseudo-pustule, umbilicated pseudo-pustule, erosion, or ulcer)

b) crusted and scabbed lesion (thick surface scale (stratum corneum), yellow serous crust, haemorrhagic crust or scab (eschar)).

c) resolved and healed lesion (lesion is no longer raised above the skin and any residual crust has resolved (desquamation)).

d) unable to classify


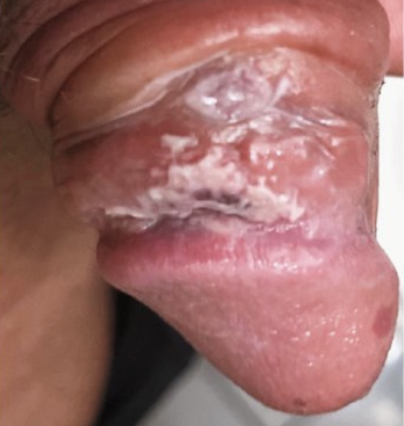


7.

a) active lesion (papules, vesicles, pustule/pseudo-pustule, umbilicated pseudo-pustule, erosion, or ulcer)

b) crusted and scabbed lesion (thick surface scale (stratum corneum), yellow serous crust, haemorrhagic crust or scab (eschar)).

c) resolved and healed lesion (lesion is no longer raised above the skin and any residual crust has resolved (desquamation)).

d) unable to classify


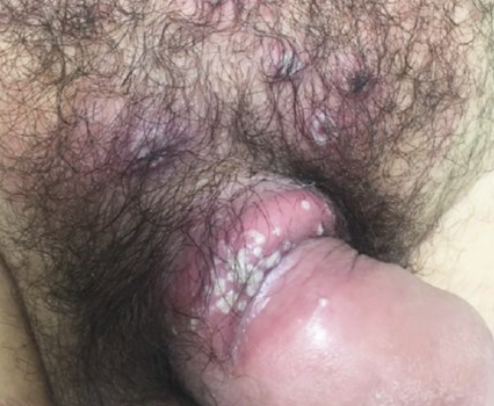


8.

a) active lesion (papules, vesicles, pustule/pseudo-pustule, umbilicated pseudo-pustule, erosion, or ulcer)

b) crusted and scabbed lesion (thick surface scale (stratum corneum), yellow serous crust, haemorrhagic crust or scab (eschar)).

c) resolved and healed lesion (lesion is no longer raised above the skin and any residual crust has resolved (desquamation)).

d) unable to classify


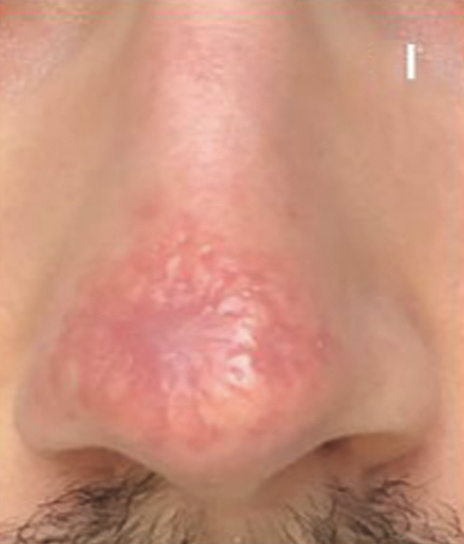


9.

a) active lesion (papules, vesicles, pustule/pseudo-pustule, umbilicated pseudo-pustule, erosion, or ulcer)

b) crusted and scabbed lesion (thick surface scale (stratum corneum), yellow serous crust, haemorrhagic crust or scab (eschar)).

c) resolved and healed lesion (lesion is no longer raised above the skin and any residual crust has resolved (desquamation)).

d) unable to classify


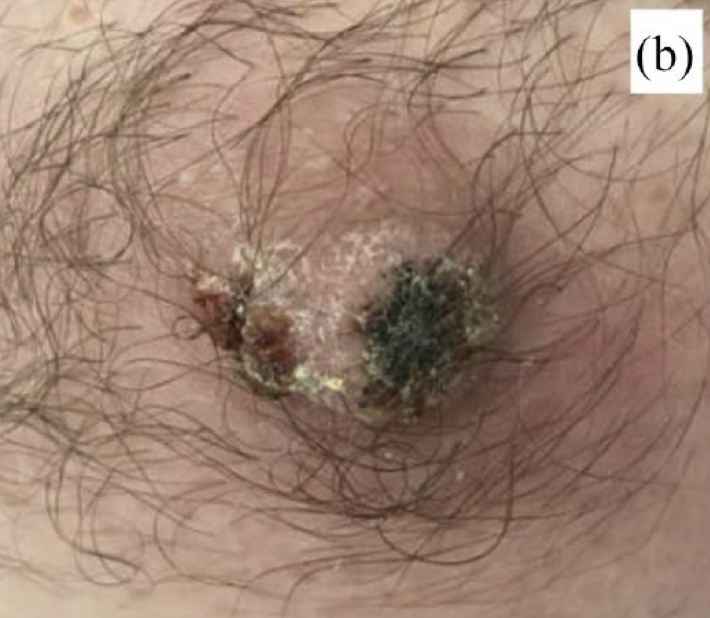


10.

a) active lesion (papules, vesicles, pustule/pseudo-pustule, umbilicated pseudo-pustule, erosion, or ulcer)

b) crusted and scabbed lesion (thick surface scale (stratum corneum), yellow serous crust, haemorrhagic crust or scab (eschar)).

c) resolved and healed lesion (lesion is no longer raised above the skin and any residual crust has resolved (desquamation)).

d) unable to classify


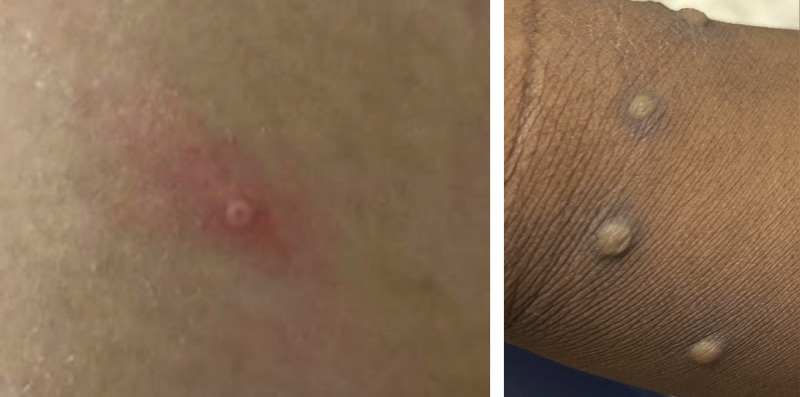


11.

a) active lesion (papules, vesicles, pustule/pseudo-pustule, umbilicated pseudo-pustule, erosion, or ulcer)

b) crusted and scabbed lesion (thick surface scale (stratum corneum), yellow serous crust, haemorrhagic crust or scab (eschar)).

c) resolved and healed lesion (lesion is no longer raised above the skin and any residual crust has resolved (desquamation)).

d) unable to classify


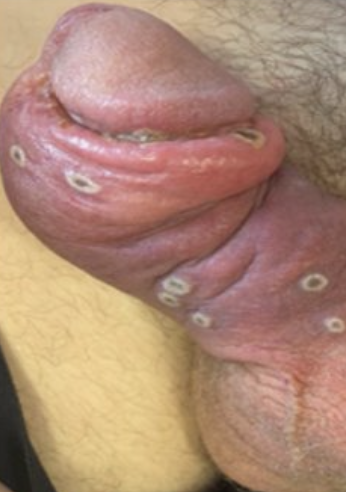


12.

a) active lesion (papules, vesicles, pustule/pseudo-pustule, umbilicated pseudo-pustule, erosion, or ulcer)

b) crusted and scabbed lesion (thick surface scale (stratum corneum), yellow serous crust, haemorrhagic crust or scab (eschar)).

c) resolved and healed lesion (lesion is no longer raised above the skin and any residual crust has resolved (desquamation)).

d) unable to classify


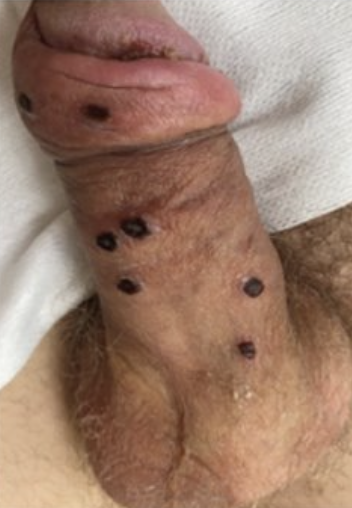


13.

a) active lesion (papules, vesicles, pustule/pseudo-pustule, umbilicated pseudo-pustule, erosion, or ulcer)

b) crusted and scabbed lesion (thick surface scale (stratum corneum), yellow serous crust, haemorrhagic crust or scab (eschar)).

c) resolved and healed lesion (lesion is no longer raised above the skin and any residual crust has resolved (desquamation)).

d) unable to classify


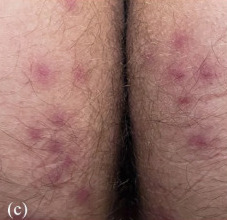


14.

a) active lesion (papules, vesicles, pustule/pseudo-pustule, umbilicated pseudo-pustule, erosion, or ulcer)

b) crusted and scabbed lesion (thick surface scale (stratum corneum), yellow serous crust, haemorrhagic crust or scab (eschar)).

c) resolved and healed lesion (lesion is no longer raised above the skin and any residual crust has resolved (desquamation)).

d) unable to classify


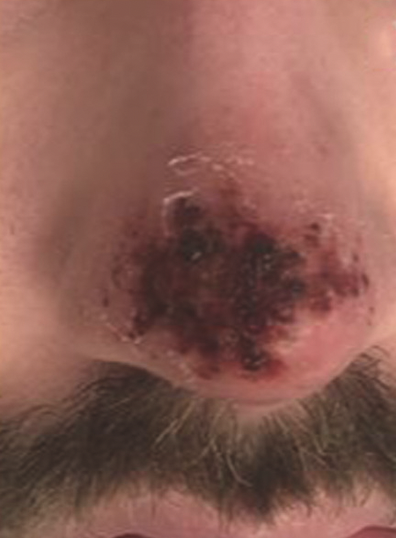


15.

a) active lesion (papules, vesicles, pustule/pseudo-pustule, umbilicated pseudo-pustule, erosion, or ulcer)

b) crusted and scabbed lesion (thick surface scale (stratum corneum), yellow serous crust, haemorrhagic crust or scab (eschar)).

c) resolved and healed lesion (lesion is no longer raised above the skin and any residual crust has resolved (desquamation)).

d) unable to classify


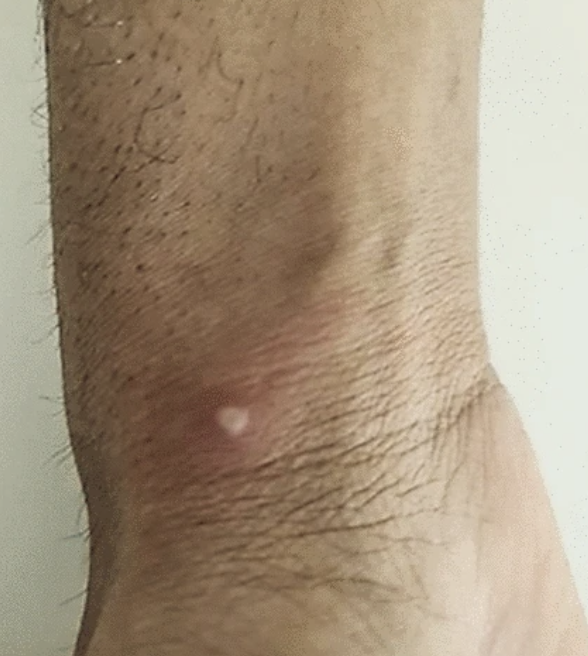


16.

a) active lesion (papules, vesicles, pustule/pseudo-pustule, umbilicated pseudo-pustule, erosion, or ulcer)

b) crusted and scabbed lesion (thick surface scale (stratum corneum), yellow serous crust, haemorrhagic crust or scab (eschar)).

c) resolved and healed lesion (lesion is no longer raised above the skin and any residual crust has resolved (desquamation)).

d) unable to classify

**
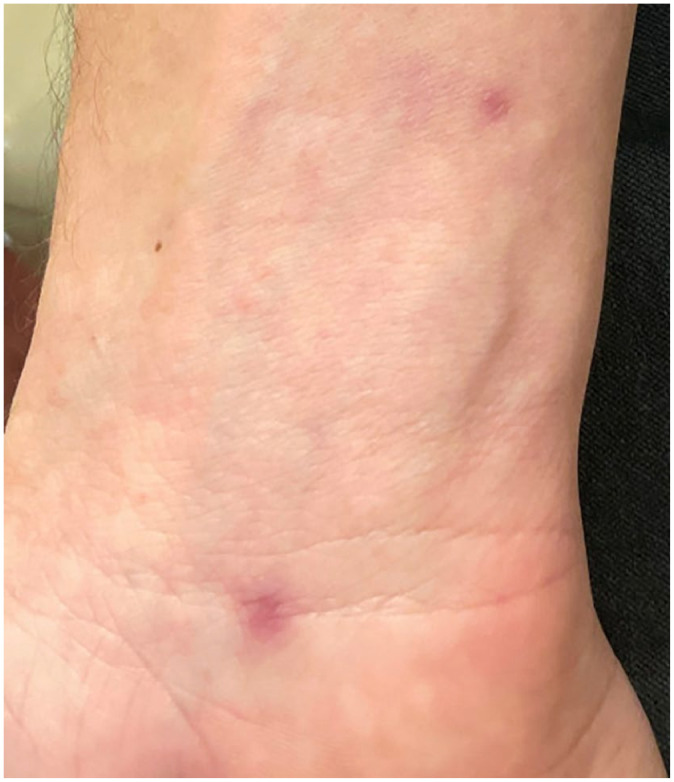
**

17.

a) active lesion (papules, vesicles, pustule/pseudo-pustule, umbilicated pseudo-pustule, erosion, or ulcer)

b) crusted and scabbed lesion (thick surface scale (stratum corneum), yellow serous crust, haemorrhagic crust or scab (eschar)).

c) resolved and healed lesion (lesion is no longer raised above the skin and any residual crust has resolved (desquamation)).

d) unable to classify


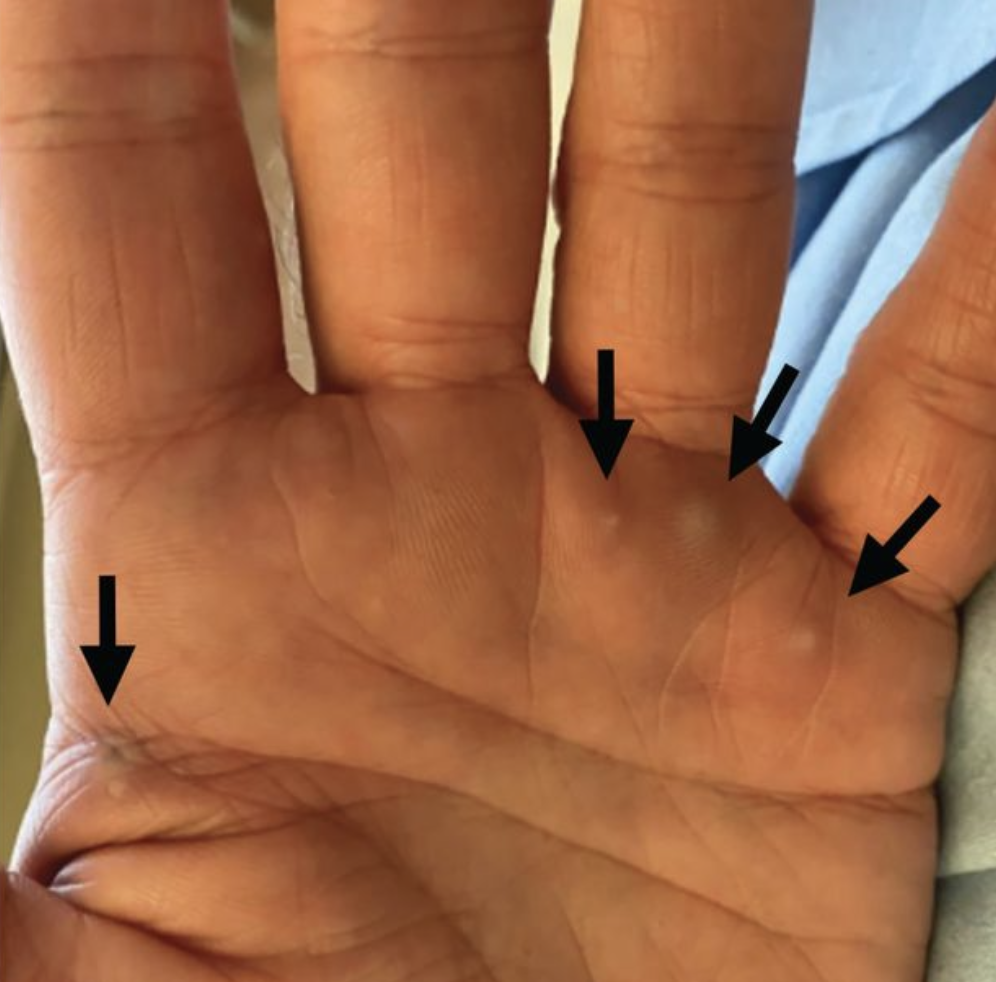


18.

a) active lesion (papules, vesicles, pustule/pseudo-pustule, umbilicated pseudo-pustule, erosion, or ulcer)

b) crusted and scabbed lesion (thick surface scale (stratum corneum), yellow serous crust, haemorrhagic crust or scab (eschar)).

c) resolved and healed lesion (lesion is no longer raised above the skin and any residual crust has resolved (desquamation)).

d) unable to classify


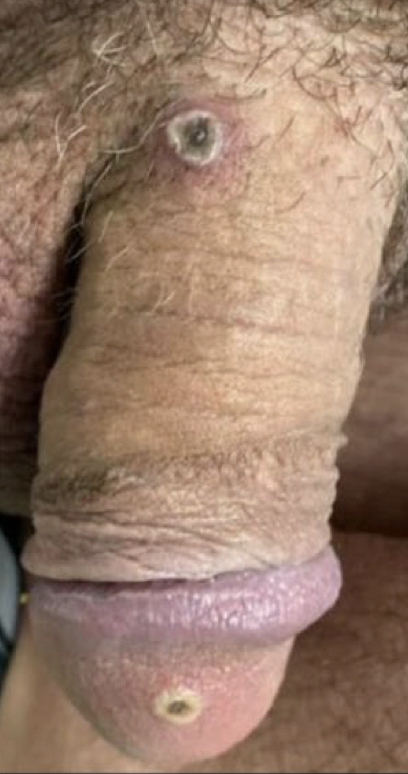


19.

a) active lesion (papules, vesicles, pustule/pseudo-pustule, umbilicated pseudo-pustule, erosion, or ulcer)

b) crusted and scabbed lesion (thick surface scale (stratum corneum), yellow serous crust, haemorrhagic crust or scab (eschar)).

c) resolved and healed lesion (lesion is no longer raised above the skin and any residual crust has resolved (desquamation)).

d) unable to classify


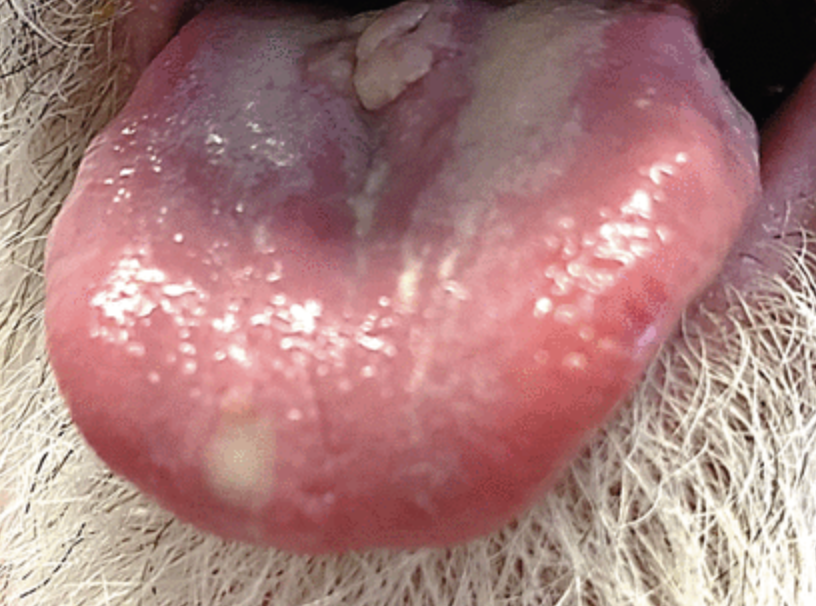


20.

a) active lesion (papules, vesicles, pustule/pseudo-pustule, umbilicated pseudo-pustule, erosion, or ulcer)

b) crusted and scabbed lesion (thick surface scale (stratum corneum), yellow serous crust, haemorrhagic crust or scab (eschar)).

c) resolved and healed lesion (lesion is no longer raised above the skin and any residual crust has resolved (desquamation)).

d) unable to classify

**Other questions:**

**What type of specialist are you?**

a) Infectious diseases

b) Genitourinary medicine

c) HIV

d) Emergency Medicine

e) General Practice

f) Dermatologist

g) Internal Medicine (no sub-specialty)

f) Other

**How many mpox patients have you seen?**

1. <5
2. 5-10
3. 10-20
4. 20-50
5. 50+

**On a scale of 1 to 10 where 1 is no confidence and 10 is complete confidence, how confident are you in assessing a mpox lesion?**

1-10 drop down list

**Do you feel that there are adequate clinical guidelines available to you for managing patients with mpox?**

Yes

No

Undecided

**What country to do you practise in (this is for description of the cohort only and will not be included in the analyses)?**

[Drop down list of countries]

Afghanistan

Albania

Algeria

Andorra

Angola

Antigua & Deps

Argentina

Armenia

Australia

Austria

Azerbaijan

Bahamas

Bahrain

Bangladesh

Barbados

Belarus

Belgium

Belize

Benin

Bhutan

Bolivia

Bosnia Herzegovina

Botswana

Brazil

Brunei

Bulgaria

Burkina

Burundi

Cambodia

Cameroon

Canada

Cape Verde

Central African Rep

Chad

Chile

China

Colombia

Comoros

Congo

Congo {Democratic Rep}

Costa Rica

Croatia

Cuba

Cyprus

Czech Republic

Denmark

Djibouti

Dominica

Dominican Republic

East Timor

Ecuador

Egypt

El Salvador

Equatorial Guinea

Eritrea

Estonia

Ethiopia

Fiji

Finland

France

Gabon

Gambia

Georgia

Germany

Ghana

Greece

Grenada

Guatemala

Guinea

Guinea-Bissau

Guyana

Haiti

Honduras

Hungary

Iceland

India

Indonesia

Iran

Iraq

Ireland {Republic}

Israel

Italy

Ivory Coast

Jamaica

Japan

Jordan

Kazakhstan

Kenya

Kiribati

Korea North

Korea South

Kosovo

Kuwait

Kyrgyzstan

Laos

Latvia

Lebanon

Lesotho

Liberia

Libya

Liechtenstein

Lithuania

Luxembourg

Macedonia

Madagascar

Malawi

Malaysia

Maldives

Mali

Malta

Marshall Islands

Mauritania

Mauritius

Mexico

Micronesia

Moldova

Monaco

Mongolia

Montenegro

Morocco

Mozambique

Myanmar, {Burma}

Namibia

Nauru

Nepal

Netherlands

New Zealand

Nicaragua

Niger

Nigeria

Norway

Oman

Pakistan

Palau

Panama

Papua New Guinea

Paraguay

Peru

Philippines

Poland

Portugal

Qatar

Romania

Russian Federation

Rwanda

St Kitts & Nevis

St Lucia

Saint Vincent & the Grenadines

Samoa

San Marino

Sao Tome & Principe

Saudi Arabia

Senegal

Serbia

Seychelles

Sierra Leone

Singapore

Slovakia

Slovenia

Solomon Islands

Somalia

South Africa

South Sudan

Spain

Sri Lanka

Sudan

Suriname

Swaziland

Sweden

Switzerland

Syria

Taiwan

Tajikistan

Tanzania

Thailand

Togo

Tonga

Trinidad & Tobago

Tunisia

Turkey

Turkmenistan

Tuvalu

Uganda

Ukraine

United Arab Emirates

United Kingdom

United States

Uruguay

Uzbekistan

Vanuatu

Vatican City

Venezuela

Vietnam

Yemen

Zambia

Zimbabwe

**References:**

 All images are used in the study under the Creative Commons Attribution Non-Commercial Licence

Images 1-2: Erez N, Achdout H, Milrot E, Schwartz Y, Wiener-Well Y, Paran N, Politi B, Tamir H, Israely T, Weiss S, Beth-Din A, Shifman O, Israeli O, Yitzhaki S, Shapira SC, Melamed S, Schwartz E. Diagnosis of Imported Monkeypox, Israel, 2018. Emerg Infect Dis. 2019 May;25(5):980-983. doi: 10.3201/eid2505.190076. Epub 2019 May 17. PMID: 30848724; PMCID: PMC6478227.

Images 3: Zlámal, Milan MD1; Bartovská, Zofia MD, PhD1; Burantová, Anna MD1; Zákoucká, Hana MD2; Jiřincová, Helena MSc3; Chmel, Martin MSc1,4; Holub, Michal MD, PhD1. Monkeypox and herpes simplex virus type 2 coinfection: Case report of perianal lesions in HIV positive patient. Sexually Transmitted Diseases: August 11, 2022

Image 4: Jang YR, Lee M, Shin H, Kim JW, Choi MM, Kim YM, Lee MJ, Kim J, Na HK, Kim JY. The First Case of Monkeypox in the Republic of Korea. J Korean Med Sci. 2022 Jul 11;37(27):e224. doi: 10.3346/jkms.2022.37.e224. PMID: 35818706; PMCID: PMC9274108.

Image 5: Vallée A, Farfour E, Zucman D. Monkeypox virus: A novel sexually transmitted disease? A case report from France. Travel Med Infect Dis. 2022 Jun 28;49:102394. doi: 10.1016/j.tmaid.2022.102394. Epub ahead of print. PMID: 35777659.

Images 6-8: Patrocinio-Jesus R, Peruzzu F. Monkeypox Genital Lesions. N Engl J Med. 2022 Jul 7;387(1):66. doi: 10.1056/NEJMicm2206893. Epub 2022 Jun 15. PMID: 35704421.

Image 9: Mileto D, Riva A, Cutrera M, Moschese D, Mancon A, Meroni L, Giacomelli A, Bestetti G, Rizzardini G, Gismondo MR, Antinori S. New challenges in human monkeypox outside Africa: A review and case report from Italy. Travel Med Infect Dis. 2022 Jun 20;49:102386. doi: 10.1016/j.tmaid.2022.102386. Epub ahead of print. PMID: 35738529.

Image 10: Costello V, Sowash M, Gaur A, Cardis M, Pasieka H, Wortmann G, Ramdeen S. Imported Monkeypox from International Traveler, Maryland, USA, 2021. Emerg Infect Dis. 2022 May;28(5):1002-1005. doi: 10.3201/eid2805.220292. Epub 2022 Mar 9. PMID: 35263559; PMCID: PMC9045429.​

Image 11: MMWR Morb Mortal Wkly Rep. 2022 Jun 10; 71(23): 764–769.

Published online 2022 Jun 10. doi: 10.15585/mmwr.mm7123e1

Images 12,13: Brown Med-Peds,. Monkeypox: The current outbreak. Brown Med-Peds Residency. (2022). Access: https://brownmedpedsresidency.org/monkeypox/

Image 14-17: Noe, S., Zange, S., Seilmaier, M. *et al.* Clinical and virological features of first human monkeypox cases in Germany. *Infection* (2022). <https://doi.org/10.1007/s15010-022-01874-z>

Images 18-20: Sukhedo, S., Tan, D., Mishra, S,. Human monkeypox: cutaneous lesions in 8 patients in Canada. *CMAJ*  (2022), 194 (38) E1323-E1327; **DOI:** 10.1503/cmaj.221087
