# Supplementary material for: Variability in clinical assessment of clade IIb mpox lesions
Source: Int J Infect Dis. 2023 Dec;137:60–2. doi: 10.1016/j.ijid.2023.10.004 (PMC10914632; doi:10.1016/j.ijid.2023.10.004)
Supplement: Supplementary file 2 [file mmc2.docx]

Appendix B – Sensitivity analysis

The robustness of the inter-rater reliability finding was assessed by recalculating Fleiss Kappa after removing question 20 from the analysis as it had a high proportion of 'unable to classify' responses (21 responses, 40%). This resulted in a Kappa value of 0.427 (p < 0.05). In addition, individual participants were excluded one at a time, and Fleiss' Kappa recalculated using the remaining participants' responses. This accounted for potential outlier participants, but the Kappa values remained in the range of 0.412 and 0.431. Lastly, we restricted the dataset to clinicians who had seen more than 20 mpox patients in their clinical practice and recalculated the inter-rater reliability for this more experienced group to assess whether the variability persisted. This minimally improved the Kappa value by 0.043 to 0.460 (p < 0.05).

The sensitivity of the inter-rater agreement was assessed by removing all ‘unable to classify’ responses (53 of 1060 responses, 9%) and recalculating the proportion of exact agreement. This analysis revealed 71.4% agreement among the participants. A breakdown of the proportion agreement per lesion with and without the sensitivity analysis is shown in Figure 1 of Appendix B.


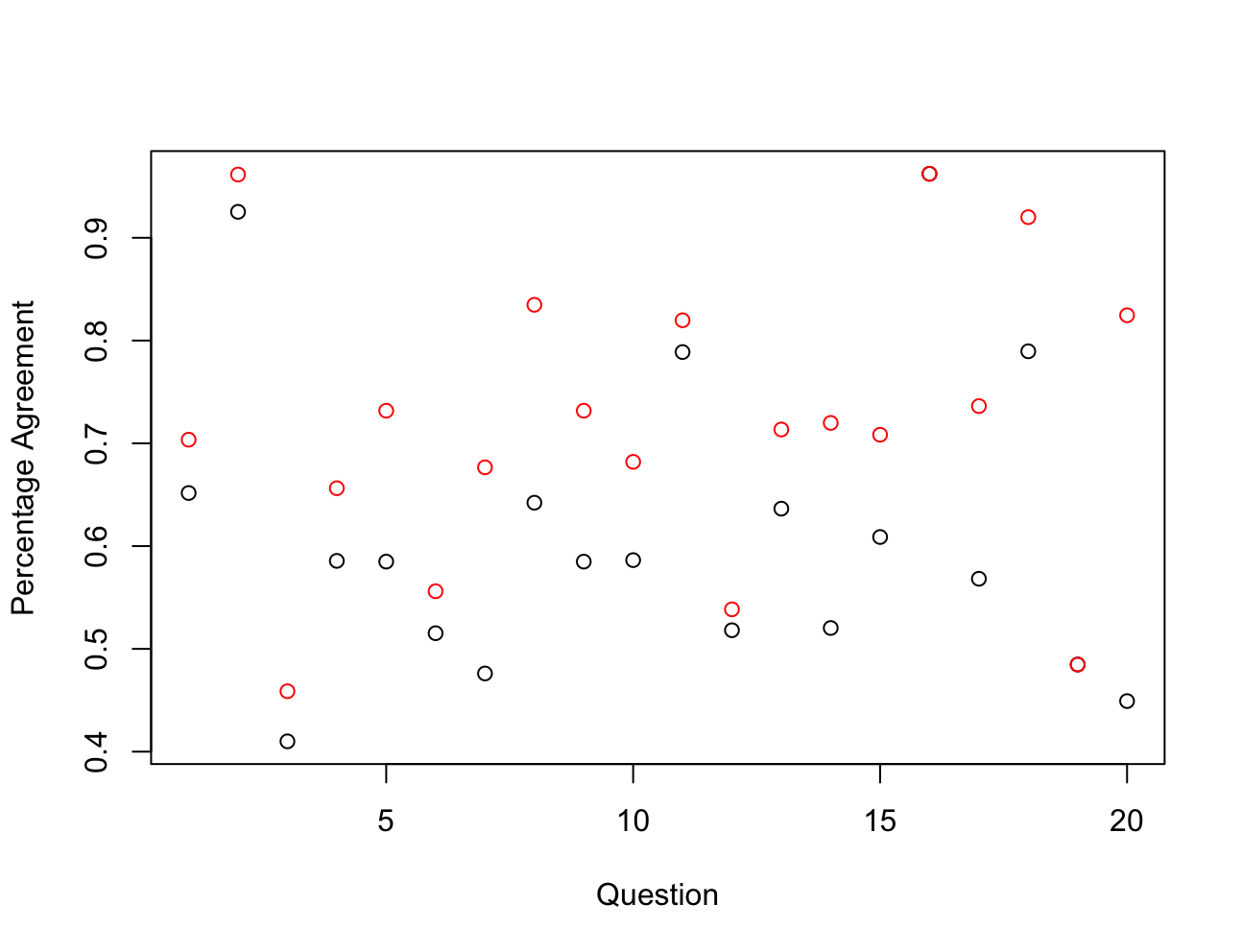


Figure 1. Proportion agreement for each question with ‘unable to classify’ responses(black) and without (red)
