# Supplementary material for: Variability in clinical assessment of clade IIb mpox lesions
Source: Int J Infect Dis. 2023 Dec;137:60–2. doi: 10.1016/j.ijid.2023.10.004 (PMC10914632; doi:10.1016/j.ijid.2023.10.004)
Supplement: Supplementary file 3 [file mmc3.docx]

Figure 1 of Appendix C. Distribution of participant's self-reported confidence assessing mpox patients on a scale of one (no confidence) to ten (complete confidence)
